# Impact of coronavirus disease 2019 (COVID-19) on healthcare-associated infections: An update and perspective

**DOI:** 10.1017/ice.2021.92

**Published:** 2021-03-12

**Authors:** Mariam A. Assi, Michelle Doll, Rachel Pryor, Kaila Cooper, Gonzalo Bearman, Michael P. Stevens

**Affiliations:** 1 Division of Hospital Medicine, Virginia Commonwealth University Health System, Richmond, Virginia; 2 Hospital Infection Prevention Program, Virginia Commonwealth University Health System, Richmond, Virginia

*To the Editor—*Infection prevention programs have been consumed by coronavirus disease 2019 (COVID-19) pandemic response efforts. There is concern that preoccupation with COVID-19 mitigation efforts might affect traditional healthcare-associated infection (HAI) surveillance and prevention operations.^1^ Evidence surrounding the impact of COVID-19 on traditional infection prevention efforts has been limited to anecdotal data and retrospective studies of highly variable quality.

We conducted 4 PubMed searches on February 4, 2021, utilizing the following search terms: “COVID-19 and healthcare associated infections,” “COVID-19 and central line associated bloodstream infections,” “COVID-19 and *Clostridioides difficile* infections” and “COVID-19 and catheter associated urinary tract infections.” In total, 43 relevant articles were retrieved; of these, only 10 reported on our outcomes of interest. The reported data were limited to retrospective cohort studies of variable quality. Key representative studies are included in Table 1.2–6 Within these limitations, these data show an increase in bloodstream infections (BSIs) in hospitals that experienced a breakdown in infection prevention best practices during COVID-19 surges while also demonstrating a decrease in *Clostridioides difficile* infection (CDI) rates.

A health system in Singapore reported a significant reduction in central-line–associated bloodstream infection (CLABSI) rates during the pandemic compared with prepandemic rates.^2^ The authors note that prior experience with severe acute respiratory syndrome (SARS) in 2003 led to the early adoption of enhanced infection prevention strategies. In contrast, other hospitals reported that COVID-19 negatively impacted infection prevention practices and observed increased BSI rates.^3,4,7^ Suboptimal nurse-to-patient ratios, barriers to accessing personal protective equipment, and barriers to performing hand hygiene were reported, as well as increased blood culture contamination rates.^3,4,7^ Data related to catheter-associated urinary tract infections (CAUTIs) are more limited. A single study from Singapore reported no change in CAUTI rates.^2^ Although not peer reviewed, the Association of Professionals in Infection Control and Epidemiology conducted a survey in which 21.4% of respondents noted an increase in CAUTIs during the pandemic and another 27.8% noted an increase in CLABSIs.^8^ Among the 3 studies included in Table 1 reporting comparative data on CDI rates, either a reduction in CDI rate or no change in CDI rate was observed.^2,5,6^

**Table 1.** Key Articles on COVID-19 and Healthcare-Associated Infections

Study Setting/Study Type/Time Comparison	Infection Being Reported	Rate/Baseline Rate	Comments
Outram campus of the Singapore Health Services group (includes the 1,785-bed Singapore General Hospital and other specialist centers in same system)^2^ Retrospective cohort study. Compared Feb 1, 2020–Aug 31, 2020 (COVID period cohort) vs Jan 2018–Jan 2020 (pre-COVID cohort)	CLABSI, CAUTI, CDI	CLABSI: COVID-period cohort with 0.20 incidents/1,000 device days (down from 0.83/1,000 device days; IRR, 0.24; 95% CI, 0.07–0.57; *P* < .05) CAUTI: No significant change in hospitalwide CAUTI rate CDI: Healthcare-facility–associated CDI did not significantly increase (3.47/10,000 patient days vs 3.65/10,000 patient days pre-pandemic, IRR, 0.95; 95% CI, 0.75–1.20; *P* = .66)	Prior experience with SARS in 2003 led to the early adoption of aggressive infection prevention bundles including universal masking, adequate PPE access. Increased CLABSI and CAUTI bundle adherence noted
Academic tertiary center in Detroit, Michigan^3^ Retrospective cohort study; Jan–May 2019 (pre-COVID cohort) vs Jan–May 2020 (COVID period cohort included patients with and without COVID-19)	CLABSI	Average monthly CLABSI rate increased to 1.7 per 1,000 central-line days (from 0.4 per 1,000 central-line days; represents a 325% increase; *P* < .01).	
Large French ICU cohort^4^ Matched case-cohort study of ICU patients; 235 patients with COVID-19 matched to 235 control patients; patients with COVID-19 included who were admitted betrween Jan 29, 2020, and Oct 3, 2020	BSI, CLABSI	35 (14.9%) ICU BSIs in COVID-19 group vs 8 (3.4%) in control group (*P* ≤ .0001) Only 10 total catheter-related BSIs detected: 8 (3.4%) in COVID-19 group vs 2 (0.9%) in non–COVID-19 group (HR, 2.5; 95% CI, 0.71–8.83; *P* = .15)	
Medical wards in hospital from Rome^5^ Retrospective analysis; Mar 1–Jun 30, 2020 compared to 2017, 2018, and 2019	CDI	COVID-19–free wards in 2020 with significantly decreased odds of healthcare-associated CDI (incidence per 100 discharges, 0.033 in 2020 compared to 0.066 in 2019; OR, 2.07; *P* = .047)	
Tertiary-care hospital in Madrid, Spain^6^ Retrospective cohort study; Mar 11–May 11, 2020, compared to same period in 2019	CDI	Incidence density of healthcare-facility–associated CDI: 2.68 per 10,000 patient days compared to 8.54 per 10,000 patient days in control period (*P* = .000257)	

Note. CLABSI, central-line–associated bloodstream infection; CAUTI, catheter-associated urinary tract infection; CDI, *Clostridiodes difficile* infection; BSI, bloodstream infection; IRR, incidence-rate-ratio; PPE, personal protective equipment; HR, hazard ratio.

Although the data are limited, at least 1 health system with robust outbreak preparedness was able to mitigate the impact of the COVID-19 pandemic and actually reported a significant reduction in CLABSIs. Conversely, multiple other health systems that reported a breakdown in infection prevention best practices reported increases in BSIs. Ultimately, our understanding of the impact of the COVID-19 pandemic on HAIs will be limited, due in part to the relaxation of mandatory reporting requirements during the pandemic.^1^ Data on healthcare-associated non–central-catheter–associated BSI rates will be particularly limited because reporting in the United States is not mandated.

Currently, the full scope of the impact of COVID-19 on HAIs is unclear. However, important opportunities exist for health systems (both on the local level and nationally) to utilize their experiences during the current pandemic to bolster infrastructure and to robustly prepare for and quickly respond to future pandemic threats.
